# A halo in the heart during coronary angiography: calcified left ventricular aneurysm with thrombus formation

**Published:** 2010-08

**Authors:** Hakan Fotbolcu, KİVİLCİM Ozden, CİHAN SENGUL, İSMET DİNDAR,, DURSUN DUMAN

**Affiliations:** Goztepe Medical Park Hospital, Division of Cardiology, Istanbul, Turkey; Goztepe Medical Park Hospital, Division of Cardiology, Istanbul, Turkey; Goztepe Medical Park Hospital, Division of Cardiology, Istanbul, Turkey; Goztepe Medical Park Hospital, Division of Cardiology, Istanbul, Turkey; Haydarpaşa Numune Training and Research Hospital, Department of Cardiology, Istanbul, Turkey

**Keywords:** left ventricle aneurysm, calcification, cardiac MRI, CT

## Abstract

A 74-year-old man presented with chest pain and dyspnoea at the cardiology outpatient clinic. His past medical history included an anterior myocardial infarction in 2008. In the coronary angiogram, a ‘halo image’ was seen right after the injection of the contrast agent, and it corresponded with the location of the left ventricular aneurysm. A calcified left ventricular aneurysm with mural thrombus was confirmed with cardiac MRI and a CT scan.

## Case report

A 74-year-old man presented with chest pain and dyspnoea at the cardiology outpatient clinic. His past medical history included an anterior myocardial infarction in 2008.

**Fig. 1.**
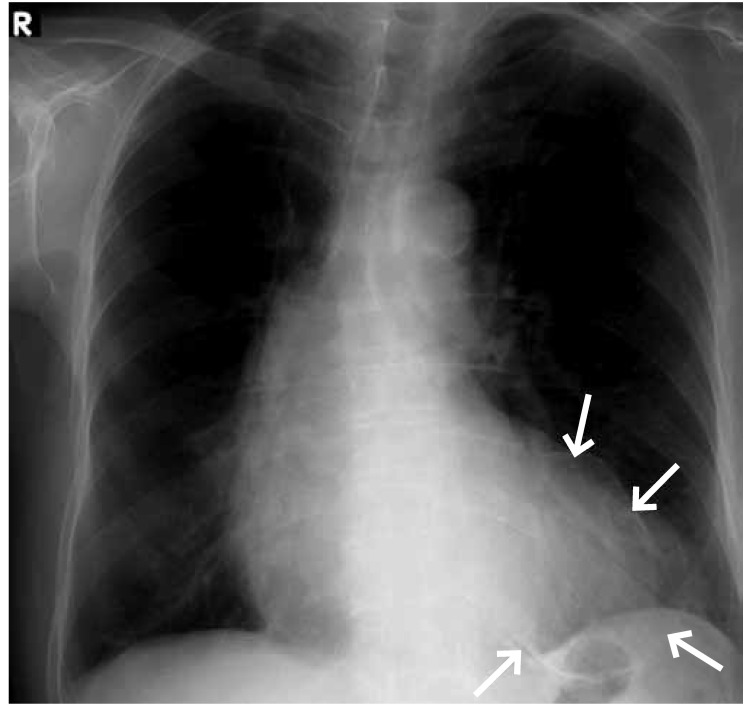
Thoracic X-ray showing an enlarged heart, and an oval-shaped calcified structure (arrows) related to a calcified antero-apical left ventricular aneurysm.

**Fig. 2.**
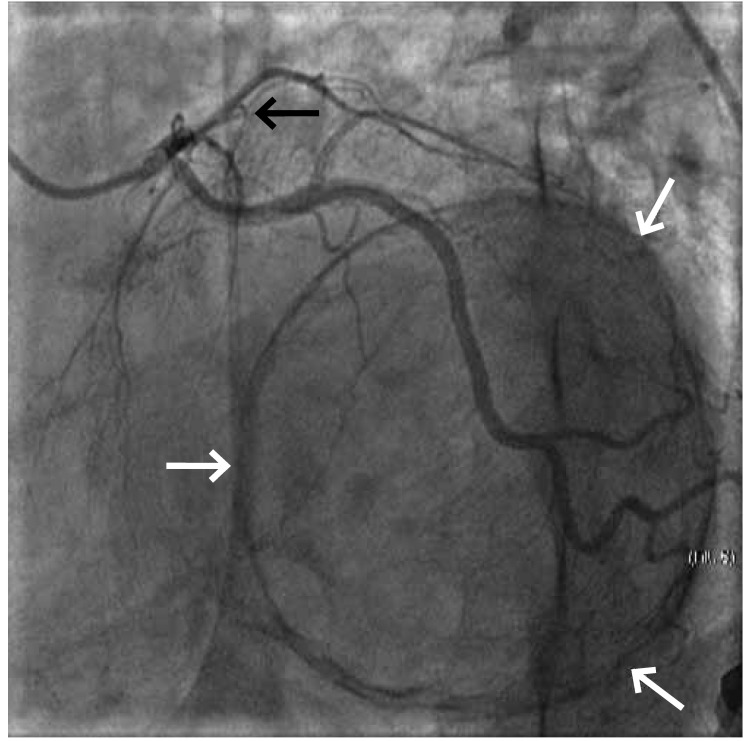
Left anterior oblique coronary angiographic image with caudal angulation showing total occlusion of the left anterior descending artery and a halo in the heart after left coronary artery injection. (The black arrow shows the point of the left anterior descending artery occlusion and the white arrows show the edge of the calcified left ventricle aneurysm.)

His ECG revealed normal sinus rhythm with poor R-wave progression in the precordial leads. A transthoracic echocardiogram demonstrated a left ventricular aneurysm with a mural thrombus and ejection fraction of 25%. The chest X-ray showed a peculiar oval calcified image related to a left ventricle aneurysm (Fig. 1). A nuclear stress test with thalium scintigraphy revealed apical mid-anterior, antero-lateral, antero-septum, infero-septum, inferior and infero-lateral scaring with minimal peri-infarct ishaemia. In the coronary angiography, a calcified aneurysm of the anterior wall similar to a huge ‘halo image’ was seen after the left main coronary artery injection, as well as a left anterior descending artery occlusion after the first diagonal branch (Fig. 2). No critical stenosis of the circumflex and right coronary artery was observed.

**Fig. 3.**
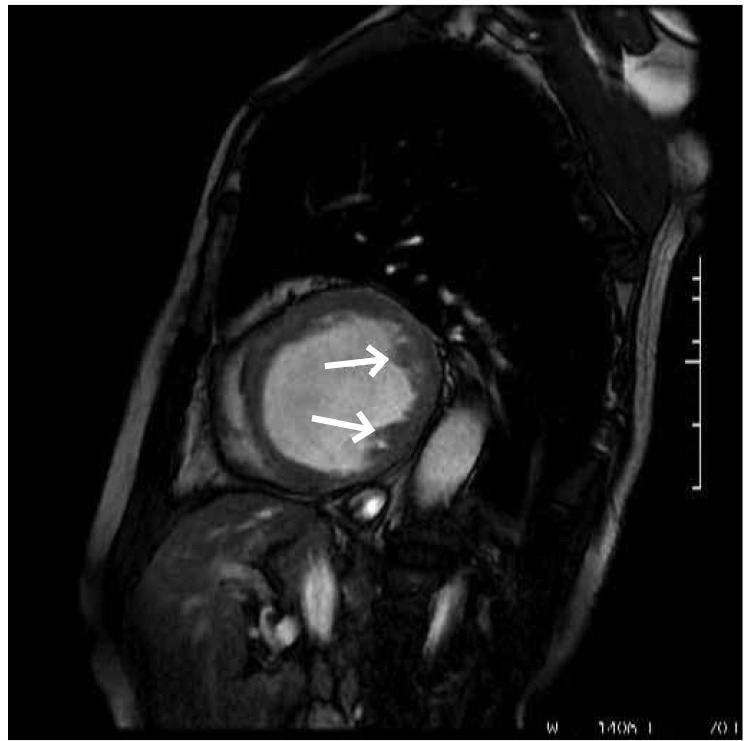
ardiac magnetic resonance imaging confirming the left ventricle aneurysm with intramural thrombus formation (the arrows show the thrombus formation in left ventricle cavity).

**Fig. 4.**
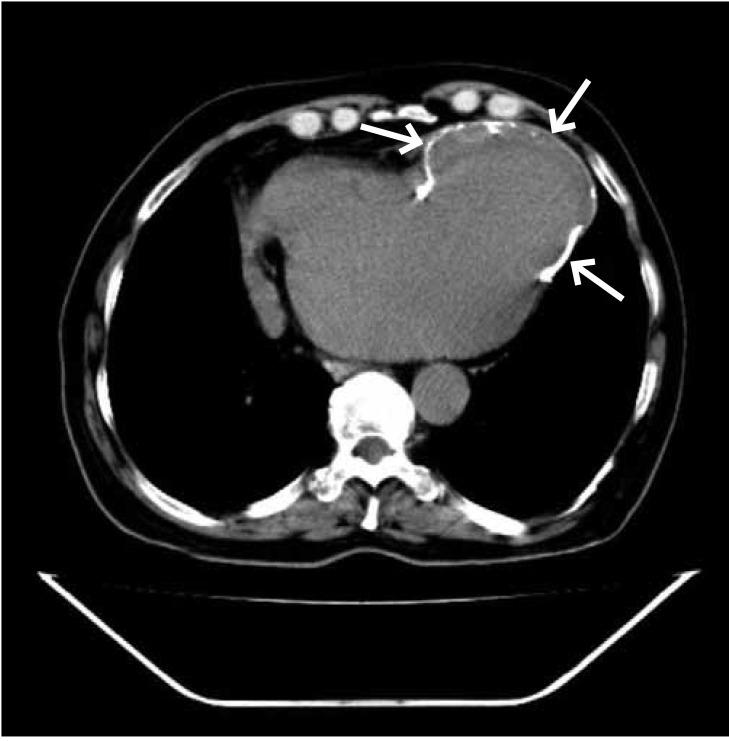
Cardiac computed tomography confirming the left ventricle aneurysm (the arrows show the edge of the calcified left ventricle aneurysm).

Left ventriculography was not performed because of the possibility of elevated left ventricular end-diastolic pressure, which might have caused the development of acute pulmonary oedema. A calcified left ventricular aneurysm with a mural thrombus was confirmed with cardiac MRI and a CT scan (Figs 3, 4). The patient was referred for coronary artery bypass surgery, however he refused to undergo this operation.

## Discussion

Left ventricular aneurysm (LVA) is a serious complication after acute myocardial infarction, and can lead to heart failure. Despite recent progress in revascularisation techniques, a large transmural myocardial infarction often results in the formation of a dyskinetic or akinetic LVA. This is followed by an enlarged ventricular cavity and abnormal ventricular shape that permits maximal conversion of tension generated by the myocardium into cavity pressure, and then congestive heart failure, arrhythmia and thrombogenesis.^1^

Curvilinear calcification at the left ventricular apex strongly suggested the presence of an aneurysm. The distribution of this calcification coincided with the typical location of left ventricular aneurysms, which are usually located at the apex and often involve the anterior and lateral walls. Trauma, cardioversion, infection and endocardial fibrosis are rare causes of coarse, amorphous myocardial calcifications, which are distinct from the fine, curvilinear calcifications of a left ventricular aneurysm.^2^

Similar previous case reports also demonstrated a calcified LVA using left ventriculography, chest X-ray, cardiac magnetic resonance imaging and computerised tomography.^3^-^5^ Interestingly, however, we showed a calcified LVA appearing like a huge halo image during coronary angiography
